# Performance of closed-loop resuscitation of haemorrhagic shock with fluid alone or in combination with norepinephrine: an experimental study

**DOI:** 10.1186/s13613-018-0436-0

**Published:** 2018-09-17

**Authors:** Nicolas Libert, Guillaume Chenegros, Anatole Harrois, Nathalie Baudry, Gilles Cordurie, Ryad Benosman, Eric Vicaut, Jacques Duranteau

**Affiliations:** 1 Laboratoire d’Etude de la Microcirculation, UMR 942, Université Paris 7, Hôpitaux Saint Louis Lariboisière, Assistance Publique-Hôpitaux de Paris, Paris, France; 2 Service d’Anesthésie-Réanimation, Hôpital d’instruction des armées Percy, Clamart, France; 3 Institut de la Vision, UMR-S 968, Sorbonne Université, Université Pierre et Marie Curie UPMC, Paris, France; 4 Service d’Anesthésie-Réanimation Chirurgicale, UMR 942, Hôpital de Bicêtre, Université Paris-Sud, Hôpitaux Universitaires Paris-Sud, Assistance Publique-Hôpitaux de Paris, Le Kremlin Bicêtre, France; 5 Unité de recherche clinique, UMR 942, Université Paris 7, Hôpitaux Saint Louis Lariboisière, Assistance Publique-Hôpitaux de Paris, Paris, France

**Keywords:** Closed-loop, Resuscitation, Haemorrhagic shock, Fluid, Norepinephrine

## Abstract

**Background:**

Closed-loop resuscitation can improve personalization of care, decrease workload and bring expert knowledge in isolated areas. We have developed a new device to control the administration of fluid or simultaneous co-administration of fluid and norepinephrine using arterial pressure.

**Method:**

We evaluated the performance of our prototype in a rodent model of haemorrhagic shock. After haemorrhagic shock, rats were randomized to five experimental groups: three were resuscitated with fluid and two with co-administration of fluid and norepinephrine. Among groups resuscitated with fluid, one was resuscitated by a physician and two were resuscitated according to two different closed-loop algorithms. Among groups resuscitated with fluid and norepinephrine, one was resuscitated by a physician and the other one by the closed-loop device. The precision of arterial pressure during the resuscitation period was assessed using rising time, time passed in the target area and performance error calculations.

**Results:**

Groups resuscitated with fluid had similar performances and passed as much time in the target area of 80–90 mmHg as the manual group [manual: 76.8% (67.9–78.2), closed-loop: 64.6% (45.7–72.9) and 80.9% (59.1–85.3)]. Rats resuscitated with fluid and norepinephrine using closed-loop passed similar time in target area than manual group [closed-loop: 74.4% (58.4–84.5) vs. manual: 60.1% (46.1–72.4)] but had shorter rising time to reach target area [160 s (106–187) vs. 434 s (254–1081)] than those resuscitated by a physician. Rats resuscitated with co-administration of fluid and norepinephrine required less fluid and had less hemodilution than rats resuscitated with fluid alone. Lactate decrease was similar between groups resuscitated with fluid alone and fluid with norepinephrine.

**Conclusions:**

This study assessed extensively the performances of several algorithms for closed-loop resuscitation of haemorrhagic shock with fluid alone and with co-administration of fluid and norepinephrine. The performance of the closed-loop algorithms tested was similar to physician-guided treatment with considerable saving of work for the caregiver. Arterial pressure closed-loop guided algorithms can be extended to combined administration of fluid and norepinephrine.

**Electronic supplementary material:**

The online version of this article (10.1186/s13613-018-0436-0) contains supplementary material, which is available to authorized users.

## Background

Haemorrhagic shock treatment requires immediate and appropriate resuscitation to preserve tissue perfusion while waiting for bleeding control. Fluid administration remains the cornerstone of the initial resuscitation of haemorrhagic shock. However, whereas fluid resuscitation is effective to restore arterial blood pressure, it may induce dilution of coagulation factors that subsequently increases blood loss [1]. Moreover, an uncontrolled increase in arterial pressure during resuscitation might split clots and induce re-bleeding [2, 3]. To avoid the potential adverse effects of fluid resuscitation, recent European guidelines on trauma haemorrhage have recommended the use of a restricted volume replacement strategy to achieve a target systolic blood pressure of 80–90 mmHg [4]. Nevertheless, targeting a tight objective of arterial pressure during the resuscitation of haemorrhagic shock is challenging for caregivers since it requires continuous attention and several interventions in a short period of time during which they have many tasks to perform concomitantly. This is even more challenging in isolated areas without specialized caregivers in case of prolonged field care or massive casualties.

To fix these issues, closed-loop (CL) resuscitation systems using hemodynamic variables have been proposed to optimize and standardize fluid administration in burn and trauma patients [5–7]. Previous automated fluid resuscitation techniques have been based on various hemodynamic targets such as mean arterial pressure, cardiac output or tissue oxygen tension [8–10]. CL resuscitation systems have been poorly evaluated in haemorrhagic shock. Comparison of their performances to physician manual resuscitation is very limited.

Because fluid therapy alone might not be sufficient to improve hemodynamic variables and excessive fluid might worsen traumatic coagulopathy, European guidelines also recommend the administration of vasopressors in case of life threatening hypotension [4]. In clinical practice, there is no clear protocol for combined administration of fluid and norepinephrine. We have developed a new CL device controller which guides simultaneous intravenous administration of fluid and norepinephrine based on systolic arterial pressure (SAP) for the resuscitation of haemorrhagic shock. In the present study, we evaluated in an extensive manner the performance of this prototype in an experimental model of haemorrhagic shock.

The first objective of this study was to compare the performances of CL resuscitation algorithms with physician manual resuscitation during early fluid resuscitation of haemorrhagic shock. The second objective was to compare the performances of CL resuscitation versus physician manual resuscitation with co-administration of fluid and norepinephrine.

We focused the present study on rat model of controlled haemorrhagic shock. Rats are particularly adapted for exploratory studies (low cost, easy accessibility and surgery). Rats have similar arterial pressure to humans and are frequently used for studies on arterial pressure-guided resuscitation [11]. Rat model is adapted for an exploratory study of arterial pressure response to diverse fluid and norepinephrine rates.

## Methods

### Animal preparation

Animals were male Wistar rats aged between 12 and 16 weeks. Rats had free access to water and feeding until they were anesthetized. Inhalation anaesthesia was performed in an induction chamber using a concentration of 2–4% of isoflurane. Anaesthesia was relayed by an intraperitoneal injection of 90 mg kg^−1^ ketamine (Imalgène 1000; Merial, Paris, France), 5 mg kg^−1^ xylazine hydrochloride (Sigma, Saint-Quentin Fallavier, France) and 50 µg kg^−1^ buprenorphine (Centravet, Maison-Alfort, France). Anaesthesia was maintained throughout the experiment with additional injections of ketamine (a quarter of the initial dose) using a peritoneal catheter. Anaesthesia efficacy was tested every 10 min using the pedal withdrawal reflex by pinching the foot pad. If the rat withdrew its leg in response to foot pad’s stimulation, then a complementary dose of anaesthesia was administered. After anaesthesia, rats were placed in the supine position on a heating blanket warmed at 38 °C. A tracheostomy was performed, and the rats breathed room air throughout the rest of the experiment. The right carotid artery was cannulated with a polyethylene catheter (PE ED 1.16 mm) which was used for exsanguination. The right femoral artery was cannulated (PE ED 0.69 mm), and this catheter was connected to a pressure transducer (Edwards Lifescience, Guyancourt, France). The right jugular vein was cannulated (PE ED 0.92 mm) to administer fluid during resuscitation. Catheters were filled with NaCl 0.9%. The pressure transducer was linked to a data acquisition system (MP30, Biopack system, Paris, France). An additional pressure transducer (Codan France, Bischwiller, France) was necessary in the CL groups. It was connected to the carotid artery catheter and linked to the CL device.

### Experimental procedure

**Fig. 1:**
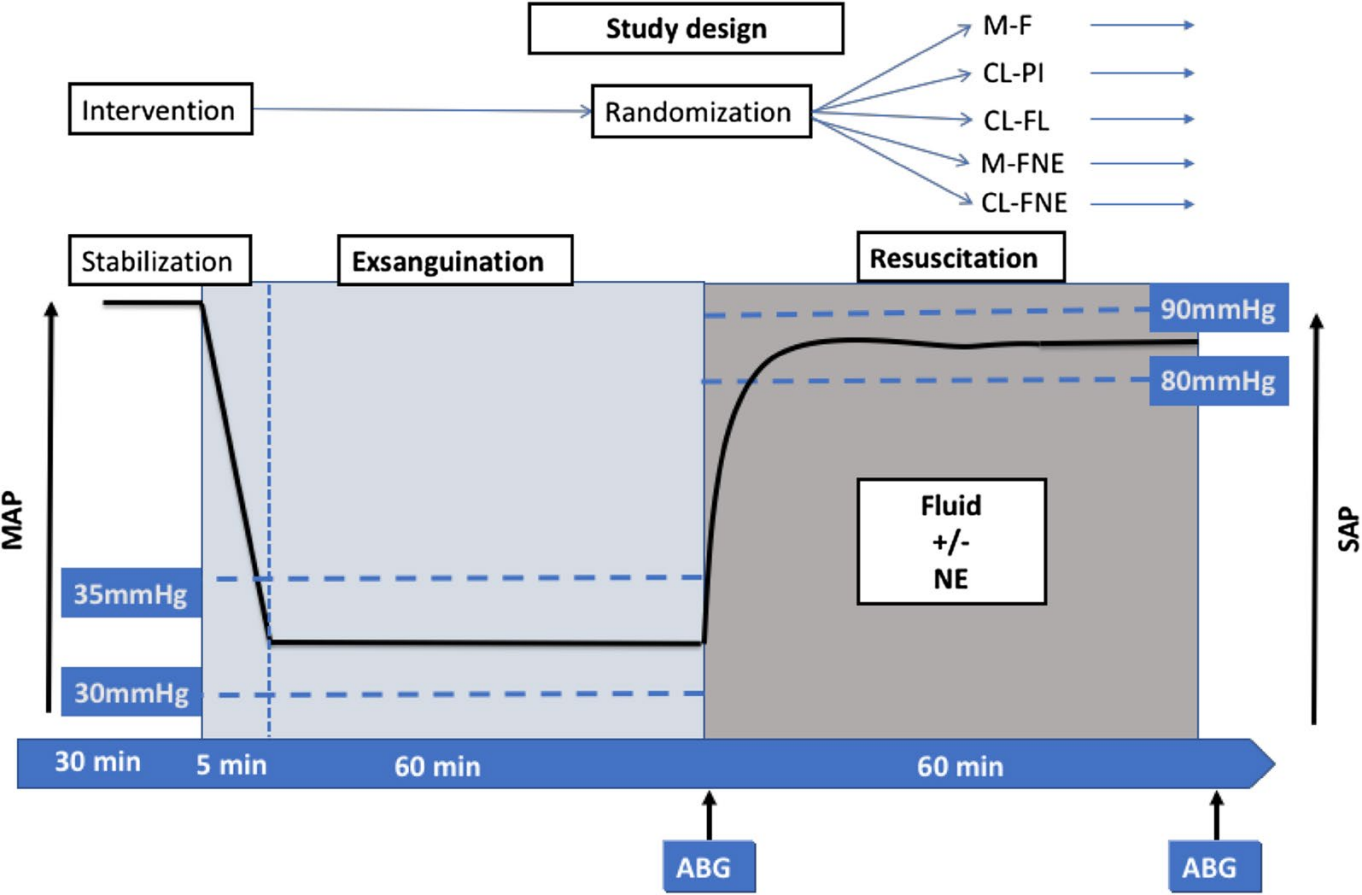
Experimental protocol. The continuous line represents the evolution of MAP during the two phases of the study (acute exsanguination followed exsanguination or transfusion to maintain MAP between 30 and 35 mmHg). Time is represented on the horizontal arrow. Each phase lasted 60 min. *ABG* arterial blood gas, *M-F* manual fluid group, *CL-PI* closed-loop PI group, *CL-FL* closed-loop FL group, *M-FNE* manual fluid and norepinephrine group, *CL-FNE* closed-loop fluid and norepinephrine group

Haemorrhagic shock was induced using a Wiggers’ model with fixed pressure [12]. Rapid exsanguination was performed for a period of 5 min to decrease mean arterial pressure (MAP) to a level of 30 mmHg. The blood withdrawn during exsanguination was stored in a heparinized syringe. MAP was maintained between 30 and 35 mmHg for 1 h by withdrawing or if necessary re-infusing blood. At the end of the exsanguination phase, rats were randomized into five groups for the resuscitation phase: three groups were resuscitated with fluid (Ringer’s Lactate solution) and two groups were resuscitated with a combined treatment of fluid and norepinephrine (Fig. 1). The resuscitation phase lasted 1 h.

Among groups resuscitated with fluid, one received standardized manual resuscitation. Fluid was administered at a fixed rate (2 mL kg^−1^ min^−1^). Infusion was started and stopped by an anaesthesiologist intensivist to target a SAP of 85 mmHg (M-F group). Two groups received automated treatment using CL based on different algorithms. The first one used a proportional–integral (PI) controller (CL-PI group) using continuous SAP acquisition to adapt infusion rate, while the second one used a fuzzy logic (FL) controller (CL-FL group) using mean SBP calculated every minute to adapt infusion rate (see Additional file 1 for a more complete description of closed-loop algorithms). To limit clot dislocation and high oscillation of arterial pressure, we limited the maximal fluid rate at 4 mL kg^−1^ min^−1^ if SAP was < 70 mmHg and at 2.5 mL kg^−1^ min^−1^ for SAP ≥ 70 mmHg.

Among groups resuscitated with combination of fluid and norepinephrine, one received manual resuscitation using a standardized protocol (M-FNE group). To achieve a progressive increase in fluid and norepinephrine, we used a protocol with alternating increase in fluid and norepinephrine. Three boluses of 10 mL kg^−1^ administered at 2 mL kg^−1^ min^−1^ of Ringer’s lactate were allowed until the maximum ceiling of norepinephrine. Norepinephrine was started at a rate of 0.1 µg kg^−1^ min^−1^ and was increased every 3 min if the target was not reached. If SAP exceeded 88 mmHg, norepinephrine rate was decreased at a rate equivalent to the mean value of the two last steps. This protocol was based on observed volumetric interactions used in clinical practice with adaptation to rat model which requires higher doses of norepinephrine. Infusion was started and stopped by an anaesthesiologist intensivist to target an SAP of 85 mmHg.

**Fig. 2:**
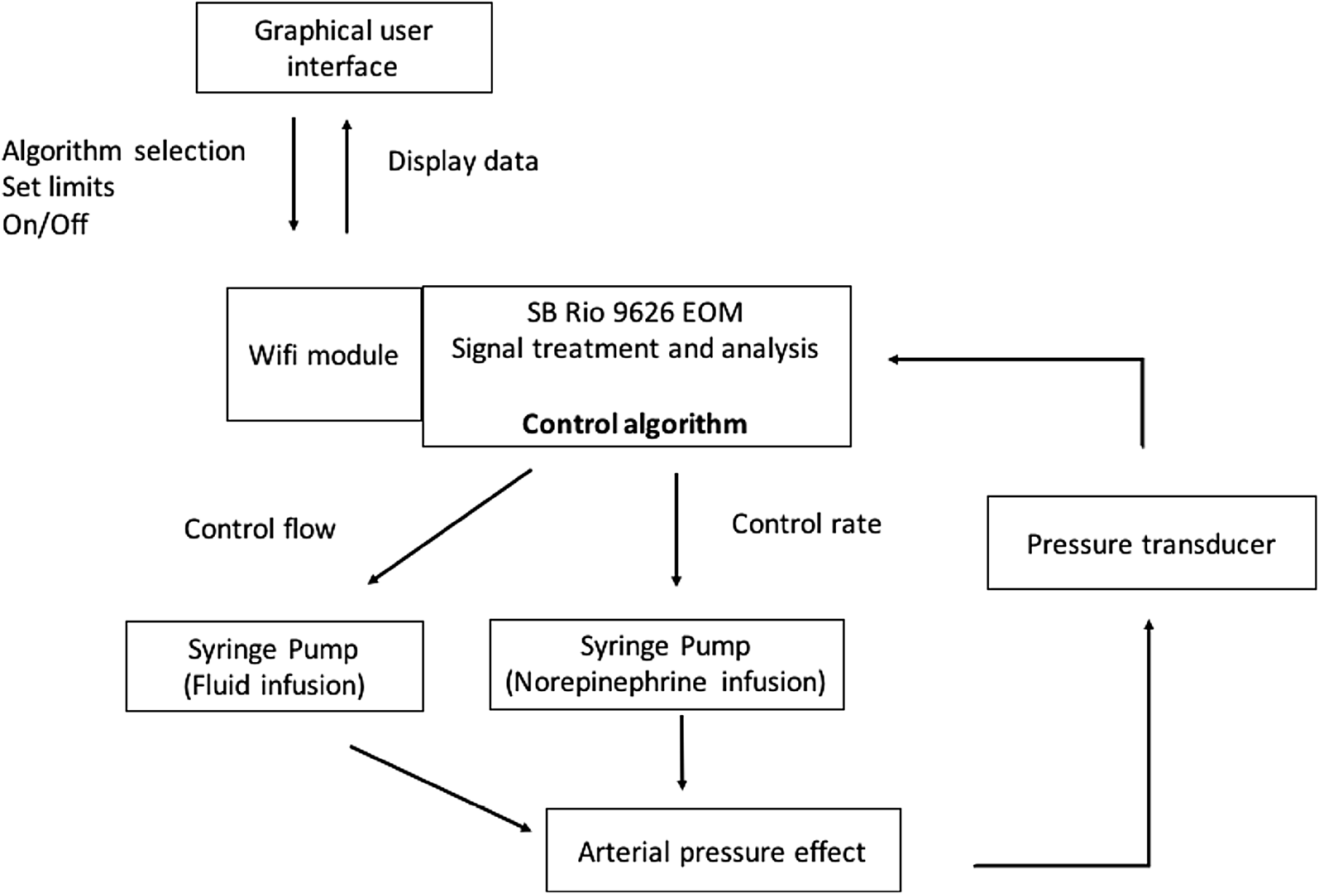
Schematic of the system set-up. The CL-FNE combined a PI regulator for fluid and a FL regulator for NE. Several conditional rules were included to mimic the physician decisions. The algorithm needed three variables: systolic arterial pressure, systolic arterial pressure error and time. During resuscitation, it calculates the ratio of fluid volume/norepinephrine to adapt therapy

The algorithm used in the group treated with automated CL combining fluid and norepinephrine (CL-FNE group) used a combination of PI and FL. The CL-FNE combined a PI regulator for fluid and a FL regulator for NE. Several conditional rules were included to mimic the physician decisions. A schematic of the system set-up is presented in Fig. 2.

Arterial pressure was extracted every 200 ms. Analysis of performance during resuscitation was divided into two phases: the rising phase of arterial pressure until 80 mmHg of SAP and the stabilization phase into the target zone. During the stabilization phase, the performance was evaluated using the following variables as described elsewhere [13]: the percentage of time passed in the target zone of SAP 80–90 mmHg, the percentage of time passed under the target zone (SAP < 80 mmHg), the percentage of time passed over the target zone (SAP > 90 mmHg), the performance error (PE), the median performance error (MDPE), the median absolute value of performance error (MDAPE), the wobble (the median absolute deviation of each PE from the MDPE) and the global score (overall performance of the system) [14].

In all groups, fluid was administered using an infusion pump (Alaris GH, Carefusion, Voisins-le-Bretonneux, France). Norepinephrine was infused using an appropriate infusion pump for small animals (Harvard Apparatus Pump 33, Harvard apparatus, Les Ulis, France).

Blood gas analyses was performed (RAPILab348EX, SIEMENS Healthcare diagnostic, Saint Denis, France) and arterial lactate (THE EDGE, ApexBio, Taiwan) and haematocrit were measured at the end of the haemorrhagic phase and immediately after the end of resuscitation using 100-µL blood gas capillary tubes.

### CL system

The CL system was based on a sbRIO 9626 EOM device (National Instruments) programmed with Labview connected to a WiFi module. The system was connected to a pressure transducer and to infusion pumps (Fig. 2). The state of the system was displayed on a personal computer in real time through a specific graphical user interface.

### Statistical analyses

Calculating the number of animals needed for the protocol is not conventional in this type of experiment since the intergroup and intra-group variance is rarely predictable [assuming the use of analysis of variance (ANOVA)]. Given the fact, on the one hand, that only large effects are sought and, on the other hand, that these experiments are complex, six–eight individuals per group are generally used. However, since the model of haemorrhagic shock is known in the literature to have significant variability, we decided to include 10 rats per group.

Normality of distribution of variables was tested by the Shapiro–Wilk test. Since the studied variables were non-normally distributed, all data are presented as median—interquartile range.

Among the three groups resuscitated with fluid, the effect of the resuscitation mode on the performance parameters and on the volume of fluid administered during resuscitation was analyzed globally using Kruskal–Wallis test. Comparisons of CL-PI or CL-FL with the M-F group were made using Dunn’s test. Among the two groups resuscitated with combined treatment, the effects of resuscitation mode were analyzed using Mann–Whitney test.

In addition, we tested the hypotheses that systemic variables such as blood gas, lactate and haematocrit measured at the end of the exsanguination period and after resuscitation can be different between groups by nonparametric two-way ANOVA for repeated measures [15].

To assess the effect of norepinephrine, we compared the groups with best performances in the presence or absence of norepinephrine by Mann–Whitney tests.

Statistical analysis was performed with GraphPad Prism 6 (GraphPad Software, San Diego, CA) and the R software (http://cran.r-project.org/) using the nparLD package.

## Results

A total of 53 rats were included in the protocol: 13 in the M-F group (three died during the early phase of resuscitation and were excluded from the analyses to avoid an artificially large difference between the measurements of performances in the group resuscitated manually and by CL devices.), 10 in the CL-PI group, 10 in the CL-FL group, 10 in the M-FNE group and 10 in the CL-FNE group. Rats weighted 348 g (318–373 g). Baseline MAP was 89 mmHg (85–99) without any difference among groups. 24.8 mL kg^−1^ (23.4–25.8) of blood, without any difference among groups, was withdrawn to achieve a target blood pressure of 30–35 mmHg during shock phase. Blood exsanguination induced a metabolic acidosis with a pH at 7.34 (7.31–7.37) and an increase in lactate concentration that peaked at 5.6 mmol L^−1^ (4.6–7.1) and a decrease in bicarbonate concentration to a bottom level of 22.3 mmol L^−1^ (21.2–23.7). Biological results are reported in Table 1.

**Table 1:**
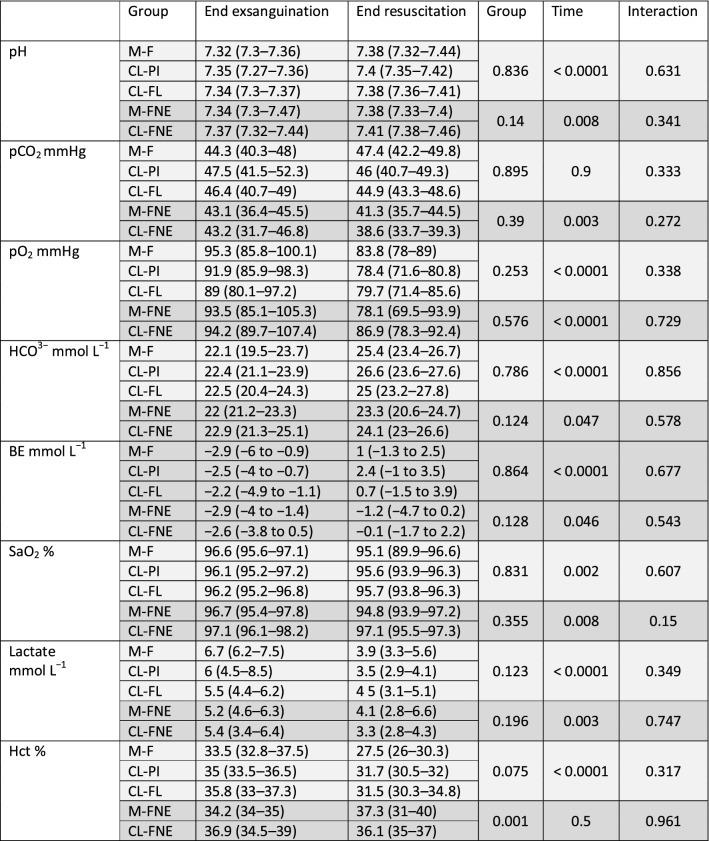
Arterial blood gas, lactate and haematocrit measurements after exsanguination and resuscitation

Results are presented for groups resuscitated with fluid alone (light grey) and for groups resuscitated with combination of fluid and norepinephrine (dark grey)

*BE* base excess, *Hct* haematocrit, *M*-*F* manual fluid, *CL*-*PI* closed-loop using a PI algorithm, *CL*-*FL* closed-loop using a FL algorithm, *M*-*FNE* manual fluid and norepinephrine group, *CL*-*FNE* closed-loop fluid and norepinephrine group

### Hemodynamic performance of resuscitation: manual (M-F group) versus CL fluid administration (CL-PI and CL-FL groups)

**Fig. 3:**
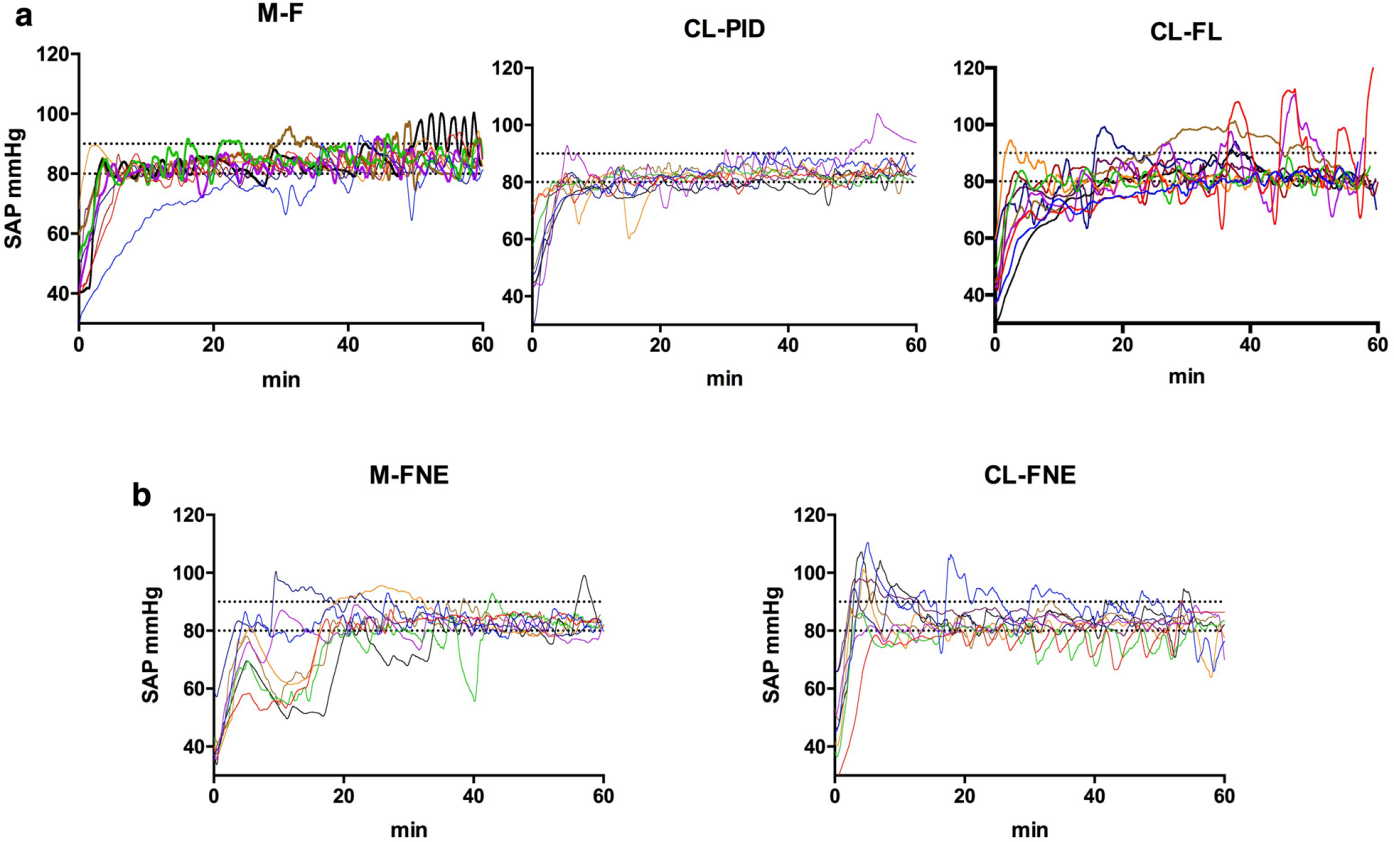
Systolic arterial pressure (mmHg) over time in groups resuscitated with fluid (**a**) and in groups resuscitated with combination of fluid and norepinephrine (**b**). *M-F* manual fluid, *CL-PI* closed-loop fluid with a PI algorithm, *CL-FL* closed-loop fluid with a fuzzy logic algorithm, *M F-NE* manual fluid and norepinephrine, *CL F-NE* closed-loop fluid and norepinephrine

SAP during resuscitation is reported in Fig. 3. As shown in Table 2, performances of CL-PI were very close to that obtained with manual resuscitation in both the time to reach the target value and the quality of the control of SAP during the maintenance period with more than 80% of time in the target area and no overshoot episode. Note, however, that the manual resuscitation required 29.5 (24.5–38) interventions to maintain SAP in the target area during the hour of resuscitation. CL-PI algorithm induced more adjustments than the manual resuscitation. The performance of CL-PI was similar to the performance of the manually resuscitated group. In contrast, CL-FL group appeared less efficient than the two other groups (Fig. 3).

**Table 2:** Performance of SAP-guided resuscitation with fluid and with combination of fluid and norepinephrine

Resuscitation	Fluid				Fluid and norepinephrine		
Groups	M-F	CL-PI	CL-FL	*p* value*	M-FNE	CL-FNE	*p* value**
Rising time (s)	226 (173–412)	252 (224–504)	529 (192–1141)	0.466	434 (254–1081)	160 (106–187)	< 0.001
Time in target area (%)	76.8 (67.9–78.2)	80.9 (59.1–85.3)	64.6 (45.7–72.9)	0.160	60.1 (46.1–72.4)	74.4 (58.4–84.5)	0.199
Time SBP < 80 mmHg (%)	21.3 (16.8–23.7)	18.6 (13.9–33.6)	29.8 (17.7–35.2)	0.481	34.5 (13.1–43.5)	12.4 (6.4–22.8)	0.139
Time SBP > 90 mmHg (%)	1.3 (0–8.7)	0 (0–2.5)	3.1 (0.1–28.1)	0.155	3.3 (0.5–18.3)	4.9 (3.3–17.7)	0.436
MDPE	− 2.6 (− 3.5 to − 1.7)	− 3.5 (− 5.3 to − 0.6)	− 3.5 (− 4.7 to − 2.1)	0.729	− 2.8 (− 4.9 to − 1.9)	− 3.5 (− 3.5 to 0.6)	0.587
MDAPE	3.6 (3.1–4.6)	3.5 (2.9–5.3)	4.7 (4.4–7.4)	0.071	4.5 (3.4–6.3)	3.5 (2.9–5.9)	0.587
Wobble	2.9 (2–3.7)	2.4 (1.8–3.5)	2.9 (2.4–7.1)	0.227	3.3 (2.9–4.2)	2.4 (1.8–4.1)	0.264
Global score	8 (6.8–12.4)	8.3 (5.5–16)	13.4 (9.4–31.9)	0.123	13 (9–23.1)	7 (5.7–17.5)	0.277
Interventions or adjustments (*n*)	29.5 (24.5–38)	1159 (792.5–1382)^†^	37.8 (19–52)	< 0.001	20 (16–31.5)	55 (30–112)	0.002

Data are presented as median [interquartile range (lower quartile to upper quartile)]

*M*-*F* manual fluid, *CL*-*PI* closed-loop using a PI algorithm, *CL*-*FL* closed-loop using a FL algorithm, *M*-*FNE* manual fluid and norepinephrine, *CL*-*FNE* closed-loop administration of fluid and norepinephrine, *SBP* systolic arterial pressure, *MDPE* median performance error, *MDAPE* median absolute value of performance error

*p value is for Kruskal–Wallis test; **p value is for Mann–Whitney test; ^†^*p* < 0.0001 in Dunn’s multiple comparisons versus M-F

Rats received similar amount of fluid in the three groups: 33.6 mL kg^−1^ (22.2–44.1), 25.5 mL kg^−1^ (23.9–45.2) and 25.5 mL kg^−1^ (23.9–45.2) in the M-F group, the CL-PI group and the CL-FL group, respectively. Fluid resuscitation improved pH and decreased lactate and haematocrit in all groups (Table 1).

### Hemodynamic resuscitation with co-administration of fluid and norepinephrine: manual (M-FNE group) versus CL administration (CL-FNE group)

SAP during resuscitation with fluid and norepinephrine is reported in Fig. 3. As shown in Table 2, CL-FNE was able to maintain SBP in the target area in almost 75% of time with very limited episodes of severe hypotension. CL-FNE had a low rising time (*p* < 0.001 vs. M-FNE group). Note that the manual resuscitation required 20 (16–31.5) interventions to maintain SAP while CL-FNE performed 55 (30–112) adjustments (*p* = 0.002).

During the 1-h resuscitation, rats of the M-FNE group received 19.9 mL kg^−1^ (13.9–21.6) of fluid and a mean dose of norepinephrine of 0.92 µg kg^−1^ min^−1^ (0.31–1.99) while those of CL-FNE group received 17.7 mL kg^−1^ (10–29.9) and a mean dose of norepinephrine of 0.61 µg kg^−1^ min^−1^ (0.21–1.39). In both groups, resuscitation improved pH and decreased lactate (Table 1).

### Effects of norepinephrine

The amount of fluid required to reach hemodynamic goal was lower in the CL-FNE group than in the CL-PI group (*p* < 0.001). This was associated to a lower dilution as attested by a more elevated haematocrit in the CL-FNE group than in the CL-PI group (36.1% vs. 31.7%, *p* = 0.011). However, pH improvement or lactate decrease was comparable to those observed in fluid resuscitation groups (Table 1).

## Discussion

The present study demonstrated the excellent performances of CL-guided resuscitation either with fluid alone or with an association of fluid and norepinephrine. CL resuscitation with co-administration of fluid and norepinephrine had a short rising time to reach the target area and excellent performance during the resuscitation phase to maintain SBP in the target area. Combination of fluid and norepinephrine reduced the fluid volume that was required to maintain SAP target in fluid resuscitation groups while achieving a similar recovery of metabolic acidosis.

### CL resuscitation and performance of CL systems

Several studies have explored the feasibility of fluid CL resuscitation for the treatment of haemorrhagic shock and severe burn [6–10, 16]. In contrast with Chaisson et al. and Rinehart et al., we have chosen to consider arterial pressure rather than cardiac output or oxygenation as the target for CL, because hemodynamic resuscitation during the initial phase of haemorrhagic shock is based primarily on arterial pressure [8, 17]. Unlike most other studies, we compared the CL performances to manual resuscitation in our model using arterial pressure-guided resuscitation since we considered that this comparison is a prerequisite for any use of such a system in patient care. It is noticeable in our study that the physician (anaesthesiologist intensivist) in charge of manual resuscitation was fully dedicated to this task contrary to clinical situations, thus possibly overestimating performances of manual resuscitation. Indeed, CL administration of fluid or fluid and norepinephrine maintained SAP in target area by means of an elevated number of manual interventions. Such a number of interventions is not feasible in clinical practice as it is not realistic to adapt SAP manually in real time so closely.

In contrast to most of previous studies in this field, we reported an extensive evaluation of CL system performances using specific variables such as MDPE, MDAPE, time in target area and global score. Varvel’s criteria are probably not sufficient to evaluate the performance of closed-loop resuscitation in haemorrhagic shock, but they are interesting to characterize the precision of arterial pressure-guided resuscitation. Only Marques et al. have reported the performance of their CL system for fluid resuscitation in a sheep model of successive haemorrhages. In their study, the percentage of time in target area was 27 ± 18%, the MDPE was − 5.6 ± 5.5%, and the MDAPE was 10.3 ± 4.5% [18].

In addition, we used high arterial data sampling in contrast to low data sampling used in some studies conducted on animals [7, 8] or human volunteers [19] that might not accurately reflect the short-term oscillations of arterial pressure.

CL algorithms using PI or FL can have specific advantages for resuscitation of haemorrhagic shock. PI is more reactive and requires continuous measurement of arterial pressure to have good performances (thus requiring an arterial line). FL is more adapted to correct coefficients when the response of the system is not known especially with discontinuous measurement of arterial pressure. In our algorithm, arterial pressure was acquired every minute in the CL-FL group. That is probably why the CL-FL appeared to be the worse. Performances of FL are appropriate when considering non-invasive blood pressure measurement and when the minimal measure of blood pressure is obtained every minute.

Whereas norepinephrine has some advantages in haemorrhagic shock, it is never used alone because it might be deleterious [20, 21]. Norepinephrine is always combined with fluid or red blood cells transfusion. Thus, we developed a CL for co-administering fluid and norepinephrine. This approach contrasts with previous works that reported studies with CL for administration of vasopressors alone or with advanced monitoring. Only sparse data are available regarding CL vasopressor therapy alone [22–25], mainly for hypotension treatment during spinal anaesthesia for caesarean section or septic shock. Uemura et al. have recently reported co-administration of fluid and norepinephrine in an animal model of endotoxin-induced shock [26]. However, their device is not adapted to haemorrhagic shock as it requires many haemodynamic invasive parameters to calculate parallel response of fluid and norepinephrine.

Combined CL treatment with fluid and norepinephrine in the unstable situation of haemorrhagic shock is highly challenging. Here, the major difficulty was to guide fluid administration and NE administration simultaneously based only on SAP. Two parallel CL systems would induce important oscillations of arterial pressure. (It is a combination of two oscillating systems, possibly creating resonance oscillations.) There are several technical solutions to solve this problem such as using different targets for the two CL, using different time points to re-evaluate rate modifications or creating fusioned variables of both systems. Here, we have used a new parameter: the ratio of fluid volume/norepinephrine rate. In other words, a fluid volume is associated to a norepinephrine upper limit. We did not simply add two CL systems, but we integrated regulation to allow each treatment to influence the other as it is the case in clinical practice. We have used a combination of PI and fuzzy logic in this CL device. CL using fluid/norepinephrine ratio and conditional rules suppressed the highly oscillating response observed in preliminary experiments using parallel CL.

Note that with CL-FNE we observed a small period of overshoot (4.9% of time > 90 mmHg) but that was limited at the initiation of the resuscitation and disappeared later in the maintenance phase. Further developments of the algorithms are likely to fix this issue.

### Effects of norepinephrine

European guidelines recommend the early use of vasopressors in case of severity to reduce fluid volume and limit dilution [4]. In addition, it is also possible to decrease or to stop vasopressor infusion while we cannot withdraw fluid that was administered.

Here, CL co-administration of fluid and norepinephrine reduced the volume of fluid required for resuscitation. This decrease in fluid reduced haemodilution, while pH improvement or lactate decrease was similar among groups resuscitated with fluid or a combination of fluid and norepinephrine. However, the optimal ratio between dose of vasopressor and volume of fluid that could improve hemodynamic with a dilution effect as low as possible, is not known.

## Limitations

Performance of the resuscitation highly depends on the shock model. Here, we used pressure-controlled haemorrhagic shock severe enough to avoid spontaneous improvement in arterial pressure. Our algorithm used continuous arterial pressure measurement, and the performance using discontinuous measurements needs to be evaluated. We used a rodent model which has a different blood volume from the one of humans, and our results need to be confirmed in large animal model.

## Conclusions

This study explored extensively the performances of several algorithms for CL resuscitation of haemorrhagic shock with fluid alone and with co-administration of fluid and norepinephrine. The performance of the CL algorithms tested was similar to optimized manual treatment by physician with considerable saving of work for the caregiver. We also demonstrated that CL algorithms can be extended to co-administration of fluid and norepinephrine in the setting of haemorrhagic shock. Closed-loop resuscitation with fluid and norepinephrine can improve personalization of care, reduce the volume of fluid required for resuscitation and limit the dilution of coagulation factors, decrease workload and bring expert knowledge in isolated areas.

## Additional file

**Additional file 1.** Description of closed-loop protocols and evaluation of performance parameters.

## Abbreviations

CL: closed-loop
MAP: mean arterial pressure
SBP: systolic blood pressure
PE: performance error
MDPE: median performance error
MDAPE: median absolute value of performance error
ANOVA: analysis of variance
