# Supplementary material for: Performance of closed-loop resuscitation of haemorrhagic shock with fluid alone or in combination with norepinephrine: an experimental study
Source: Ann Intensive Care. 2018 Sep 17;8:89. doi: 10.1186/s13613-018-0436-0 (PMC6141407; doi:10.1186/s13613-018-0436-0)
Supplement: Supplementary file 1 — Additional file 1. Description of closed-loop protocols and evaluation of performance parameters. [file 13613_2018_436_MOESM1_ESM.docx]

**Description of closed-loop protocols.**

A closed-loop device integrates the response of the system by feedback mechanisms. It is a feedback controller that automatically adapts the parameters of the controlled devices (output) according to the continuous or discontinuous measurement of different physical parameters or biological quantities (input). The fed back signal objective is to reduce the signal error (which is the difference between the parameter target value and the actual parameter target value) and improve stability. In the present study, fluid and norepinephrine infusion rates were controlled according to the evolution of blood pressure. The closed loop controller used two types of algorithms: PID and Fuzzy Logic.

In a PID controller, the correction performed to reduce error is based on proportional, integral and derivative terms. The mathematical function of the control input to the plant is expressed as following:

$$u\left( t \right)=K_{p}.e^{(t)}+ K_{i}.\int_{0}^{t} e^{(\tau)}d\tau+ K_{d}.\frac{de^{(t)}}{dt}$$

Where $K_{p}$, $K_{i}$ and $K_{d}$ denote the coefficients for the proportional, integral and derivative terms respectively. In the present study, $K_{p}=0.1$. $T_{i}=3$ and $T_{D}=0$. Therefore, the controller was a PI controller.


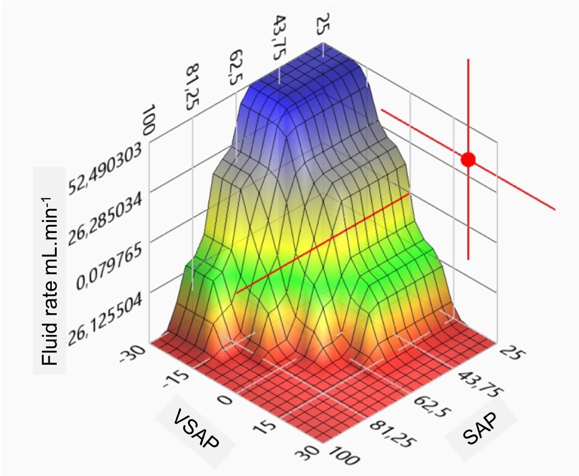
A Fuzzy Logic controller is designed to reproduce human reasoning. Contrary to the classical logic in which states or classifications are binary (either true or false), in fuzzy logic, they can be both true and false at the same time, with some degree of belonging to each of these two situations (partially true). Fuzzy variables describe vague concepts, such as a fast car, hot weather, weekend days. These variables are most often qualitative and used to describe quantities (large, small, medium, far, close, etc.). The reasoning on these "uncertain" linguistic variables will make it possible to manipulate knowledge in descriptive natural language. In the first step, variables are transformed by fuzzification into fuzzy variables. Then, the input variables of a fuzzy controller are mapped by sets of membership function (the Fuzzy Set). Membership functions are processed according to specific rules (for example: if MAP is very low, then norepinephrine rate is largely increased). The combined results are converted into a specific control output value. Usually, a coefficient map is built with the different variables used in the controller. Here is an example of the coefficient map of a fluid regulator:

In this case, the infusion rate is determined by the systolic arterial pressure (SAP) and the variation of the systolic arterial pressure (VSAP).

**Closed-loop control of combination of fluid and norepinephrine.**


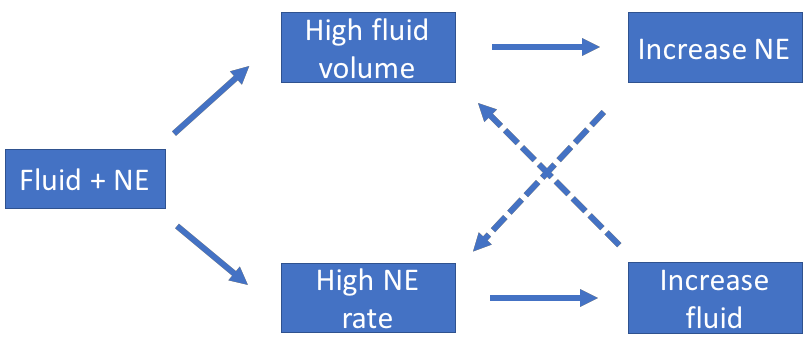
In clinical practice, fluid and norepinephrine are not clearly protocolized. Norepinephrine is used early, usually at a low rate (around 0.1 µg.kg^-1^.min^-1^). When the patient receives significant volume of fluid norepinephrine is increased. When the norepinephrine rate becomes high, another fluid challenge is administered, and so on. This observation was the starting point of the development of the algorithms used for CL-FNE.

Concept of clinical use of fluid and norepinephrine for hemorrhagic shock.

NE: norepinephrine

The CL-FNE combined a PID regulator for fluid and a FL regulator for NE. Several conditional rules were included to mimic the physician decisions. The algorithm needed 3 variables: systolic arterial pressure, systolic arterial pressure error and time. During resuscitation, it calculates the ratio of fluid volume/norepinephrine to adapt therapy.


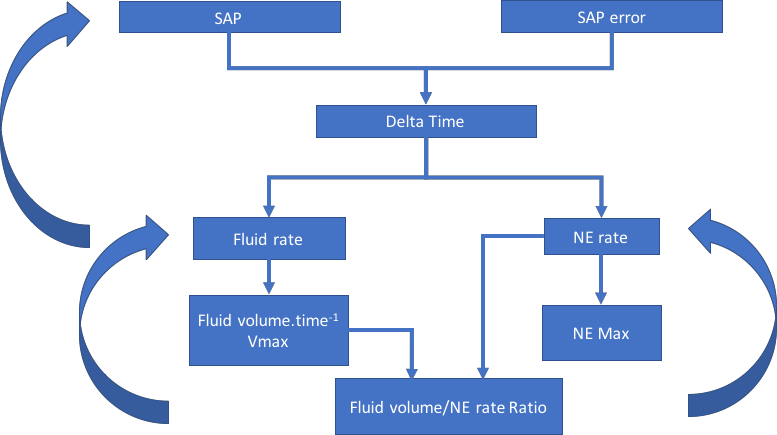
CL-FNE algorithm.

SAP: systolic arterial pressure. SAP error: systolic arterial pressure error. Delta time: time between modifications.

**Evaluation of performance parameters.**

**Performance error (PE) is defined as the difference between each measured value and the**

**target value. PE is expressed as a percentage of the target value**

$${PE}_{ij}=\frac{{meSAP}_{ij}-{tarSAP}_{ij}}{{tarSAP}_{ij}}\times100$$

meSAP: measured systolic arterial pressure. tarSAP: target systolic arterial pressure. i: subject number. j: j^th^ measurement of observation period. n: total number of measurements during the resuscitation period.

**Median performance error (MDPE) reflects the bias of the controller and is defined**

**as the median of all values of the performance error. It represents the direction of the prediction error.**

$${MDPE}_{i}=median\{{PE}_{ij}, j=1,\ldots,N_{i}\}$$

i: subject number. j: j^th^ measurement of observation period

**Median absolute value of performance error (MDAPE) reflects the inaccuracy of the controller and is defined as the median of the absolute values of the performance error. It represents the size of the performance error.**

$${MDAPE}_{i}=median\left\{ \left| {PE}_{ij} \right|, j=1,\ldots,N_{i} \right\}$$

i: subject number. j: j^th^ measurement of observation period

**Wobble is a measure of the variability of the performance error around MDPE. It is defined as the median absolute deviation of performance error from MDPE.**

$${WOBBLE}_{i}=median\left\{ \left| {PE}_{ij}-{MDPE}_{i} \right|, j=1,\ldots,N_{i} \right\}$$

i: subject number. j: j^th^ measurement of observation period

**The Global score (GS) characterizes the overall performance of the system.**

$${GS}_{i}=\frac{\left( {MDAPE}_{i}+{WOBBLE}_{i} \right)}{\% Time in target area}$$

i: subject number
